# Stroke Outreach in an Inner City Market: A Platform for Identifying African American Males for Stroke Prevention Interventions

**DOI:** 10.3389/fneur.2015.00133

**Published:** 2015-06-15

**Authors:** Anjail Zarinah Sharrief, Brenda Johnson, Victor Cruz Urrutia

**Affiliations:** ^1^Department of Neurology, University of Texas Medical School at Houston, Houston, TX, USA; ^2^Department of Neurology, Comprehensive Stroke Center, The Johns Hopkins Hospital, Baltimore, MD, USA; ^3^Department of Neurology, Johns Hopkins University School of Medicine, Baltimore, MD, USA

**Keywords:** knowledge, disparity, race, stroke, prevention

## Abstract

**Background:**

There are significant racial disparities in stroke incidence and mortality. Health fairs and outreach programs can be used to increase stroke literacy, but they often fail to reach those at highest risk, including African American males.

**Methods:**

We conducted a stroke outreach and screening program at an inner city market in order to attract a high-risk group for a stroke education intervention. A modified Framingham risk tool was used to estimate stroke risk and a 10-item quiz was developed to assess stroke literacy among 80 participants. We report results of the demographic and stroke risk analyses and stroke knowledge assessment.

**Results:**

The program attracted a majority male (70%) and African American (95%) group of participants. Self-reported hypertension (57.5%), tobacco use (40%), and diabetes (23.8%) were prevalent. Knowledge of stroke warning signs, risk factors, and appropriate action to take for stroke symptoms was not poor when compared to the literature.

**Conclusion:**

Stroke outreach and screening in an inner city public market may be an effective way to target a high-risk population for stroke prevention interventions. Stroke risk among participants was high despite adequate stroke knowledge.

## Introduction

Although stroke incidence has decreased over time, it remains higher among African Americans compared to White Americans (1). Studies have shown that while the majority of people do not have a basic understanding of stroke, stroke literacy is poorer among African Americans (2, 3). Health fairs are often used to screen and counsel individuals at risk for stroke; however, these venues tend to attract an older more health conscious population (4). Screening programs often fail to reach younger people at high risk, especially African Americans and males (1, 2, 4).

In February 2008, we began a program of community outreach at an inner city market that serves a majority African American population. The Northeast Market Project (NEMP) was designed to serve as a platform for addressing barriers for risk factor control in a high-risk community. In 2010, we began a study to assess the effect of education on participants’ stroke risk factor profiles. In this paper, we report the results of the baseline knowledge assessment.

## Materials and Methods

IRB approval was obtained from Johns Hopkins Hospital IRB. Participants were recruited among passersby in the market who agreed to have their blood pressure measured. Participants who met inclusion criteria for the educational intervention signed informed consent. Inclusion criteria included age 18 or older and willingness to provide a phone number. The exclusion criterion was refusal to sign consent.

After signing consent, participants were given a 10-item multiple-choice quiz to assess stroke knowledge. The quiz items were compiled from the National Stroke Association self-assessment quiz and from true/false or multiple-choice questions created by study investigators (Supplementary Material) (5). Questionnaire content was similar to prior stroke literacy studies (2, 3, 6–12).

Stroke risk was calculated using a modified Framingham stroke risk profile (mFSRP) assessment tool, which did not include left ventricular hypertrophy assessment (13). This tool requires information about stroke risk factors, including prior diagnosis of hypertension, diabetes mellitus, coronary artery disease, as well as current tobacco use. The tool differs from the standard Framingham Stroke Risk Profile because it does not include an assessment of LVH, and therefore, may underestimate actual stroke risk. Self-reported data about the presence of risk factors were used to calculate risk factor prevalence. The Maryland Behavioral Risk Factor Surveillance Screen data were used to compare the prevalence of risk factors in participants to prevalence in the population of Maryland (14). Automated and calibrated blood pressure cuffs (HEM-711DLX OMRON Healthcare Bannockburn, IL, USA) were used to obtain a single blood pressure measure. All participants received counseling on risk factor modification.

STATA version 12 was used for data analysis. (*Stata Statistical Software: Release 12*. College Station, TX, USA) (15). Significant *p*-values are considered <0.05. *T*-tests and tests of proportion were used to compare demographic variables by gender. Linear regression was used to evaluate relationships between demographic variables and baseline score. We only report baseline knowledge in this report.

## Results

Among 81 individuals recruited for the study, 1 participant refused further participation after initial screening. In the first 6 months of the project, 131 were screened, and 66 signed consented. Baseline demographic data did not differ between those screened and those participating. Demographic data were available for 80 participants (Table 1). Males represented 70% of the sample and 95% of the participants identified as African American. More than 95% of participants lived in Baltimore city.

**Table 1:** **Baseline characteristics**.

	Mean (SD)^a^		
	Males (*n* = 56)	Females (*n* = 24)	Total (*n* = 80)
Age (years)	51.8 (11.1)	50.5 (11.8)	51.5 (11.2)
Age range	26–82	22–72	22–82
Race, no (%)			
African American	53 (94.6%)	23 (95.8%)	76 (95%)
Other	3 (5.4%)	1 (4.2%)	4 (5%)

*Demographic characteristics of consented participants in the Northeast Market Project stroke knowledge study*.

*^a^Except noted*.

The prevalence of self-reported hypertension was 57.5% (CI 46.4, 68.6). The prevalence was slightly higher in men (60.7%) than women (50%) but this difference was not significant (*p* = 0.374). Among participants without prior diagnosis of hypertension, 35% had blood pressure in the hypertensive range and 44% had blood pressure in the pre-hypertensive range.

The overall prevalence of tobacco use was also high in this group at 40% (CI 29.0, 51.0) and was similar for men and women. Diabetes prevalence was 23.8% (CI 14.2, 33.3), and coronary artery disease prevalence was 17.5% (CI 9.0, 26.0). Atrial fibrillation based on patient report was present in 7.5% of the sample (CI 1.6, 13.4). The average 10-year risk of stroke was 8.56% (CI 6.8, 10.3) and approximately 30% of participants had greater than or equal to ten percent 10-year risk of stroke according the modified Framingham risk factor score.

The prevalence of self-reported risk factors among participants was compared to the overall population of Maryland using Behavioral Risk Factor Surveillance Screen data from 2009 and 2010 (14). Hypertension, tobacco use, diabetes, and coronary artery disease were each significantly more prevalent in the Northeast Market participants compared to the population of Maryland (Table 2).

**Table 2:** **Prevalence of risk factors and comparison to Maryland Behavioral Risk Factor Surveillance System**.

	Northeast Market	Maryland BRFSS	
Hypertension (95% CI)	57.5% (46.4, 68.6)	36.8% (35.7, 37.8)	*p* = 0.0001
Tobacco use	40% (29.0, 51.0)	14% (13.3, 14.7)	*p* < 0.0001
Diabetes	23.8% (14.2, 33.3)	12.3% (11.6, 12.9)	*p* < 0.002
Coronary artery disease	17.5% (9.0, 26.0)	5.4% (4.94, 5.86)	*p* < 0.0001

*Self-reported risk factors among participants compared to self-reported Maryland resident data from Maryland Behavioral Risk Factor Surveillance System 2009–2010*.

Participants answered 66.2% (SD 19.5%) of all questions correctly. Table 3 lists percentages correct for each question with confidence intervals. Questions are provided in Supplementary Material. All three signs and symptoms of a stroke were identified by 77.5% of participants. All three more common risk factors of hypertension, tobacco use, and diabetes were identified by 80% (CI 71.1, 89.0) of participants. Conversely, all three less common risk factors – sleep apnea, irregular heart rate (atrial fibrillation), and abdominal obesity – were identified by 58.6% of participants (CI 47.7, 69.8). The majority (87.5%, CI 80.1, 94.9) of NEMP participants would call 911 for symptoms of stroke.

**Table 3:** **Stroke knowledge assessment**.

Questions	Percent correct (95% CI) *n* = 80
Q1. Where in the body does a stroke occur?	57.5% (46.4, 68.6)
Q2. Which of the following represent(s) a stroke?	77.5% (68.1, 86.9)
Q3. There is no treatment for stroke once it occurs. T or F^±^	71.2% (61.1, 81.4)
Q4. Which are risk factors for stroke?	80% (71.1, 89.0)
Q5. Which of these is used to prevent stroke?	73.8% (63.9, 83.6)
Q6. What should you do if you experience stroke symptoms?	87.5% (80.1, 94.9)
Q7. Stress is number one risk factor for stroke. T or F^±^	10% (3.2, 16.7)
Q8. Which of the following decreases risk of stroke?	55% (43.9, 66.1)
Q9. Stroke is the #3 cause of death in Maryland. T or F^±^	86.3% (78.5, 94)
Q10. Which of the following is associated with increased risk of stroke?	58.6% (47.7, 69.8)

*^±^T or F, true or false*.

*See Supplementary Material for full questionnaire*.

*Multiple-choice questions included in the baseline knowledge assessment and proportion of subjects answering correctly with 95% confidence intervals (CI)*.

Linear regression analysis did not reveal associations between overall score and age (*p* = 0.473), race (*p* = 0.946), gender (*p* = 0.752), hypertension (*p* = 0.720), tobacco use (*p* = 0.935), heart disease (*p* = 0.437), diabetes (*p* = 0.794), or 10-year stroke risk (*p* = 0.292).

## Discussion

This sample of African American, mostly male, Baltimore city residents was relatively young and had a high risk of stroke, as suggested by prevalence of self-reported hypertension, tobacco use, diabetes, and coronary artery disease and by stroke risk score. These results suggests that conducting a screening program in an inner city market is a sound approach to addressing barriers to risk factor control, which may contribute to the higher risk of stroke in young African American males.

Stroke knowledge assessments across different racial and ethnic populations have shown varying levels of awareness of stroke etiology, risk factors, and symptoms (2–4, 7–12, 16). Different methods have been used to assess stroke knowledge including telephone surveys, and open and close-ended in-person interviews and questionnaires (2). In general, stroke knowledge has been shown to be poorer among racial minorities and individuals of lower socioeconomic status. We used close-ended multiple choice and true/false questions to assess stroke knowledge. Because of the open-ended format of questions asked in prior studies, it is difficult to make comparisons to the literature. However, we are able to make some comparisons to the few studies with close-ended format.

One question assessed knowledge of stroke symptoms, and 77.5% (CI 68.1, 86.9) of NEMP participants answered correctly that weakness, slurred speech, and vision loss were all symptoms of stroke. The correct identification of these symptoms varies between 35% and 95% in the literature (3, 8–10, 12). Two questions assessed knowledge of major stroke risk factors. Among NEMP participants, 80% (71.1, 89.0) answered that hypertension, diabetes, and tobacco use were all risk factors for stroke. In the literature, correct identification of these risk factors varies between 42% and 94%, with hypertension most often identified in close-ended questions (3, 11, 12). Ninety percent of participants incorrectly answered that stress is the “number one” risk factor for stroke. A number of studies have demonstrated that stress is identified as a major stroke risk factor among African Americans (7, 11, 12). Only one question assessed knowledge of action to take for stroke signs or symptoms, and 87.5% of NEMP participants answered that they would call 911 immediately. Other studies have shown that approximately 40–90% of participants would call 911 (2, 3, 6, 7, 9–12).

Considering prior studies, the baseline knowledge assessment showed that for most questions, participants’ knowledge was not poor, but similar or better than that reported in the literature. A number of factors could contribute to better knowledge than expected in this population, including the presence of the NEMP itself. Furthermore, the participants may have previously been exposed to healthcare professionals because of their risk factors, and this exposure may have contributed to their health literacy. Finally, these individuals could have also participated in other Baltimore-based cardiovascular and hypertension studies (17). The finding that stroke literacy is not poor in this group suggests that knowledge alone is not sufficient to impact behaviors associated with stroke risk factors.

A number of factors may explain poor risk factor control and high stroke risk despite adequate stroke knowledge. These include socioeconomic conditions impacting access to medical care as well as access to healthy foods and safe spaces for exercise. Self-efficacy or confidence in one’s ability to modify behaviors and change norms is also important for health behavior change (18). Other barriers at the patient, provider, and system level may all interact to impact risk factor control (19). Factors such as stress and discrimination may also contribute to the knowledge/risk discordance noted in this population (20). Interventions to bridge the gap between knowledge and behavior will be a focus of future studies. We will also assess individual-level and environmental factors that impact behaviors.

This study has several limitations. Because of the use of a convenience sample, the group may not be representative of the population. The close-ended format of questions may have been leading, thereby overestimating stroke knowledge. Socioeconomic status is associated with stroke knowledge, and this was not assessed. Finally, while the knowledge questionnaire has not been validated, questions were to similar to those used in prior literacy studies.

The major strength of this study is that we have identified an approach to reaching young African American males. The study of this group is critical because the high incidence of stroke in African American men contributes to the disparities in stroke incidence observed among African Americans compared to White Americans. This is a group in which preventive efforts may have a large impact in reducing disparities in stroke incidence associated with race. Using this platform, we can design and test behavioral interventions for stroke risk factor control.

## Author Contributions

AS contributed to the conception and design of the work, the acquisition, analysis, and interpretation of data, and of drafting the work; gave final approval of the version to be published; and agreed to be accountable for all aspects of the work in ensuring that questions related to the accuracy or integrity of any part of the work are appropriately investigated and resolved. BJ contributed to conception and design of the work, the acquisition of data for the work, and revised it critically for important intellectual content; gave final approval of the version to be published; and agreed to be accountable for all aspects of the work in ensuring that questions related to the accuracy or integrity of any part of the work are appropriately investigated and resolved. VU contributed to the conception and design of the work, the acquisition and interpretation of data, and revising the work critically for intellectual content; gave final approval of the version to be published; and agreed to be accountable for all aspects of the work in ensuring that questions related to the accuracy or integrity of any part of the work are appropriately investigated and resolved.

## Conflict of Interest Statement

The authors declare that the research was conducted in the absence of any commercial or financial relationships that could be construed as a potential conflict of interest.

## Supplementary Material

The Supplementary Material for this article can be found online at http://journal.frontiersin.org/article/10.3389/fneur.2015.00133/abstract
