# Supplementary material for: Stroke Outreach in an Inner City Market: A Platform for Identifying African American Males for Stroke Prevention Interventions
Source: Front Neurol. 2015 Jun 15;6:133. doi: 10.3389/fneur.2015.00133 (PMC4467175; doi:10.3389/fneur.2015.00133)
Supplement: Supplementary file 1 [file presentation_1.pdf]

Stroke Knowledge Questionnaire

Select the best answer for each question. The best answer may be “all of the above.”

1. Where in the body does a stroke occur?
    a. Heart
    b. Brain
    c. Kidneys
    d. Lungs

2. Which of the following could represent a stroke?
    a. Sudden loss of vision
    b. Weakness on one side of the body
    c. Sudden onset of slurred speech
    d. All of the above

3. There is no treatment for stroke once it occurs.
    a. True
    b. False

4. Which of the following increase(s) a person’s risk of stroke?
    a. High blood pressure
    b. Smoking cigarettes
    c. Diabetes (high blood sugar)
    d. All of the above

5. Which of the following medications is/ are used to prevent stroke?
    a. Aspirin
    b. Tylenol
    c. Valium
    d. All of the above

6. What should you do if you experience signs or symptoms of stroke?
    a. Call your primary care doctor
    b. Take an aspirin and wait until symptoms resolve
    c. Take a nap
    d. Call 911 immediately

7. Stress is the number one risk factor for stroke
    a. True
    b. False

8. Which of the following can a person do to decrease their risk of stroke?
    a. Stop smoking cigarettes
    b. Stop drinking coffee

   c. Avoid exercise
   d. All of the above

9. Stroke is the 3rd leading cause of death in Maryland.
   a. True
   b. False

10. Which of the following is associated with increased risk of stroke
    a. Sleep apnea
    b. Large waist size
    c. Irregular heart rate
    d. All of the above
